# Perineural steroid injections around ilioinguinal, iliohypogastric, and genitofemoral nerves for treatment of chronic refractory neuropathic pain: A retrospective study

**DOI:** 10.1080/24740527.2017.1403846

**Published:** 2017-12-15

**Authors:** Rajinikanth Sundara Rajan, Anuj Bhatia, Philip W. H. Peng, Allan S. Gordon

**Affiliations:** a Department of Anaesthesia and Pain Medicine, University of North Midlands NHS Trust, Staffordshire, United Kingdom; b Department of Anesthesia and Pain Management, University of Toronto, University Health Network–Toronto Western Hospital, Toronto, Ontario, Canada; c Institute of Health Policy Management and Evaluation, University of Toronto, University Health Network–Toronto Western Hospital, Toronto, Ontario, Canada; d Division of Neurology, University of Toronto, Wasser Pain Management Center, Mount Sinai Hospital, Toronto, Ontario, Canada

**Keywords:** ilioinguinal nerve, iliohypogastric nerve, nerve block, abdominal wall, neuropathic pain, injection, steroids

## Abstract

**Background**: Perineural local anaesthetic and steroid injections around ilioinguinal (II), iliohypogastric (IH), and genitofemoral (GF) nerves are often performed to treat chronic refractory neuropathic pain in the lower abdomen and groin, but there is a lack of published data on outcomes of these interventions.

**Aims**: The objective of this retrospective study was to evaluate analgesic outcomes of ultrasound-guided II, IH, and GF nerve blocks in patients with chronic neuropathic pain in the lower abdominal wall and groin.

**Methods**: Analgesic outcomes were assessed at 6 weeks after injections and patients were classified as “responders” if the numerical rating scale for pain score reduced by 30% or more. Variables analyzed for impact on outcomes included demographics, intensity of pain and duration, etiology, dose of opioid, presence of anxiety, depression, and diabetes mellitus.

**Results**: In this cohort of 54 patients with severe baseline pain who had failed to receive analgesic benefit from recommended first- and second-line medications for neuropathic pain, 30 patients had history of surgery and 24 had pain secondary to visceral inflammatory pathologies. Twenty-five (46.3%) patients were identified as responders. A majority of the patients in this cohort had pain for more than one year. There was a higher incidence of diabetes mellitus in nonresponders compared to responders but the difference was not significant (14% and 0%, respectively; *P* = 0.115).

**Conclusions**: Ultrasound-guided perineural steroids can ameliorate chronic refractory abdominal wall and groin neuropathic pain in patients who have failed to respond to conventional medical management at 6 weeks after the procedures.

## RÉSUMÉ

**Contexte:** L’anesthésie locorégionale périneurale et les injections de stéroïdes à proximité du nerf ilio-inguinal (II), du nerf ilio-hypogastrique (IH) et du nerf génito-fémoral (GF) sont souvent utilisées pour traiter la douleur neuropathique chronique réfractaire dans le bas-ventre et dans l’aine, mais peu de données ont été publiées sur les résultats de ces interventions.

**But:** L’objectif de cette étude rétrospective était d’évaluer les résultats analgésiques du blocage des nerfs II, IH et GF guidé par ultrasons chez des patients souffrant de douleur neuropathique chronique dans la paroi du bas-ventre et dans l’aine.

**Méthodes:** Les résultats analgésiques ont été évalués six semaines après les injections et les patients ont été classés « répondeurs » si le score obtenu sur l’échelle numérique pour la douleur avait diminué de 30 % ou plus. Les variables analysées pour leur effet sur les résultats comprenaient les données démographiques; l’intensité et la durée de la douleur; l’étiologie; le dose d’opiacés, la présence d’anxiété; la dépression; et le diabète sucré.

**Résultats:** Parmi cette cohorte de 54 patients souffrant d’une douleur sévère au départ pour lesquels les médications de première et de deuxième ligne recommandées pour la douleur neuropathique n’avaient pas été bénéfiques, 30 patients avaient une histoire de chirurgie et 24 avaient des douleurs secondaires à des pathologies inflammatoires viscérales. 25 patients (46,3 %) ont été identifiés comme répondants. La majorité des patients de cette cohorte ressentaient de la douleur depuis plus d’un an. Il y avait une plus grande incidence de diabète sucré chez les non-répondeurs comparativement aux répondeurs mais la différence n’était pas significative (14 % et 0 % respectivement; p = 0,115).

**Conclusions:** Les stéroïdes périneuraux guidés par ultrasons peuvent améliorer la douleur neuropathique chronique réfractaire dans la paroi abdominale et dans l’aine chez les patients qui n’ont pas répondu à la prise en charge médicale conventionnelle six semaines après les procédures.

## Introduction

Chronic lower abdominal wall and groin pain is a frequent presentation in pain clinics and up to 25% of adults have abdominal pain at any one time.^1^ It is often encountered following surgery on the abdominal and pelvic wall and it accounts for approximately 10% of patients with chronic idiopathic abdominal pain.^2^ The etiology and pathology tend to be complex and multifactorial. Antecedent surgery is commonly associated with the development of persistent pain in the abdomen and genital regions.^3^ Macrae defined chronic postsurgical pain (CPSP) as pain developed after surgery and of at least two months duration, after exclusion of other caused of pain including the pre-existing pain.^4^ Hernia repairs are the most frequently performed operations worldwide and it is accepted that CPSP is the most common and serious long-term complication after inguinal hernia repair.^5–7^ Damage to ilioinguinal (IL), iliohypogastric (IH), and genitofemoral (GF) nerves during the intraoperative period can contribute to neuropathic pain after lower abdominal surgeries^8^ and a neuropathic component of pain is estimated to be present in up to 50% of patients with chronic pain after inguinal hernia repair.^9^ Inflammatory conditions of the abdomen and pelvis (e.g., interstitial cystitis, endometriosis, inflammatory bowel disease) can also be a cause of neuropathic pain in the abdominal wall and groin from entrapment of nerves innervating the overlying skin in fibrosis and scar tissue.^1^,^10,11^

Patients with chronic lower abdominal wall and groin pain should be managed with a multimodal approach based on the biopsychosocial model of pain, with pharmacological therapies often being the first step.^12^ However, pharmacologic treatment with medications such as gabapentinoids and antidepressants to relieve neuropathic pain is often ineffective with responder rates varying from one in three to one in seven.^13^ These medications also have significant adverse effects, including sedation, drowsiness, edema, and cognitive impairment, that limit increases in dose and compliance. In patients with refractory neuropathic pain, perineural injections of steroids around the II, IH, and GF nerves are often performed for diagnostic and therapeutic purposes.^14^ Prognostication of response to surgical neurectomy is another indication for these interventions.^15^ The rationale for this therapy is that steroids prolong the duration of analgesia by stabilizing neuronal membranes and also by inhibiting the synthesis and release of proinflammatory mediators.^16,17^

There is some evidence for analgesic efficacy of injections of perineural steroids around IH and II nerves, but the published studies are heterogeneous with methodological limitations.^18–21^ Existing literature is unable to provide a reliable estimate of analgesic outcomes of these procedures. There is a paucity of reliable data about the nature of pain syndromes and characteristics of patients who undergo these interventions. This retrospective study on data from patients at our two clinics was conducted with the primary objective of evaluating analgesic outcomes of II, IH, and GF perineural injections with local anaesthetics (LAs) and steroids at 6 weeks after the interventions for patients with chronic refractory neuropathic pain in the lower abdominal wall and the groin. The secondary objective was to identify variables associated with responders to this intervention.

## Methods

This retrospective cohort study was conducted at the Interventional Pain Clinic of Toronto Western Hospital, University Health Network and the Wasser Pain Management Centre at Mount Sinai Hospital in Toronto, Canada, following research ethics board approvals (REB No.s 13-7004-AE and 14-0038-C, respectively). Strengthening of Reporting of Observational Studies in Epidemiology guidelines were followed during reporting of this retrospective study.^22^ Data were analyzed from a database of adult patients (aged ≥ 18 years) who received ultrasound-guided local anaesthetic and steroid injections around II, IH, and GF nerves between January 1, 2009, and July 31, 2013. Pain centers at both of these hospitals are large, tertiary-level multidisciplinary pain clinics. Around 450 patients receive ultrasound-guided injections of perineural LA and steroids per year for neuropathic pain secondary to various etiologies and refractory to noninterventional management (pharmacological options, psychological treatments, and physical therapy). Patients with abdominal wall and groin pain are part of this cohort, and these patients are referred to the two clinics by surgeons, gynecologists, urologists, gastroenterologists, internists, and family physicians.

### Patient selection

Patients were identified from the clinical database of perineural procedures and their details were cross-matched against procedural lists of the two pain clinics. Electronic medical records of patients eligible for inclusion in the study (as defined below) were also reviewed. All patients had a diagnosis of refractory neuropathic pain of moderate or severe intensity based on the following criteria: pain of new onset following surgery on the abdomen or groin or in association with inflammation of abdominal or pelvic viscera; pain present in distribution of II and IH nerves with or without pain in distribution of GF nerves; pain described using one or more neuropathic pain descriptors selected by patients from the Short Form McGill Pain Questionnaire; abnormal sensory findings on physical examination; and refractory pain defined on the basis of “rule-of-four” used at our centers (intensity of pain ≥ *4* on the 11-point Numerical Rating Score [NRS] for pain, pain persisting for *four or more months* after surgery, no analgesic benefit from a trial of at least one medication in each of the *four main classes* for treatment of neuropathic pain [gabapentinoids, tricyclic antidepressants, serotonin and norepinephrine uptake inhibitors, opioids]) or adverse effects resulting in discontinuation of medications.^23^ All patients had undergone extensive assessment by medical and surgical specialists to rule out treatable etiologies for ongoing pain. Though patients in our study cohort had pain from surgical and nonsurgical etiologies, the presentation was distinctly neuropathic and we considered our cohort as homogenous in terms of the pathology (entrapment of the nerves in scar tissue), presentation, and treatment protocols. This therapeutic approach is also endorsed by others, including the International Association for Study of Pain’s Special Interest Group on Neuropathic Pain.^13^

### Injection technique

All perineural IH, II, and GF nerve injections were performed by staff pain physicians experienced in ultrasound-guided perineural injections. Sonosite M-Turbo (Sonostie Fujifilm Inc, Bothell, WA) with a linear transducer (13–6 MHz) was used for ultrasound guidance and injections were performed as per the technique described by others and our group.^24,25^ A standard protocol for perineural injections is used at our clinics. Injectate consisted of 6 mL of 0.25% bupivacaine and 60 mg of methylprednisolone acetate (Depo-Medrol, Pfizer Cananda Inc., Kirkland, Quebec, Canada) around IH and II nerves at locations superior and posterior to the anterior–superior iliac spine and 4 mL of 0.25% bupivacaine and 20 mg of methylprednisolone acetate around GF nerves in the spermatic cord or round ligament (in patients who had pain in distribution of this nerve). The steroid preparation was a single-dose, preservative-free vial and its use was off-label. Accuracy of the injections was confirmed by demonstration of numbness in the area of sensory distribution of targeted nerves 15 min after the procedure. None of the patients in this cohort had repeat injections prior to the follow-up visit at 6 weeks.

### Measurement of outcomes and selection of variables

Patients who had 30% or more reduction in the pain NRS score compared to preintervention baseline values were categorized as responders and the rest were nonresponders. This dichotomization was based on Initiative on Methods, Measurements, and Pain Assessment in Clinical Trials recommendations.^26^ The primary outcome of interest was to establish the proportion of responders following the interventions. The secondary outcome of this study was to identify variables associated with analgesic response. Information was extracted from the database and medical records for variables to be analyzed, including age, sex, data related to pain (description, duration and location of pain, preprocedural NRS score, presence of pain in other areas of the body), nature of surgery and any postoperative complications, nature of inflammatory visceral condition and related complications, psychological comorbidities (presence of anxiety and depression as diagnosed by Hospital Anxiety and Depression Scale),^27^ diagnosis of diabetes mellitus, preprocedural pharmacological treatment (use of any neuropathic pain medication entered as a yes/no response, dose of opioids as daily oral morphine equivalents in milligrams), and any complication related to the perineural injections. Among these variables, based on clinical rationale, we considered the following likely to have an impact on our outcome of interest: age, sex, etiology, intensity and duration of pain, dose of opioids, presence of anxiety, depression, and diabetes mellitus.

Adverse effects over the 6-week follow-up period were also recorded. These included reports of thinning of skin or necrosis at injection site; reports of impaired glycemic or blood pressure control in patients with history of diabetes mellitus and hypertension, respectively; history of fractures during the postprocedure period; and evidence of impaired immunity as manifested by recurrent infections or impaired wound healing.

### Statistical analysis

The data collected from the study contained continuous and categorical variables. Continuous data were examined for normality of distribution using the Kolmogorov-Smirnov test. Continuous data with normal distributions were summarized as means and standard deviations, and data with nonnormal distributions were summarized as medians and interquartile ranges. Categorical data were summarized as numbers and percentages. Variables with normal distribution were analyzed using *t*-tests, and those with nonnormal distributions were analyzed using Wilcoxon rank sum tests. All *t*-tests were two-sided and unpaired. For *P*-values of *t*-tests, pooled values were used for equality of variances and Satterthwaite values were used for unequal variances. Categorical variables were analyzed using chi-square test (or Fisher’s exact test when any cell had an expected count of less than five). Exploratory analyses were performed to analyze the influence of various domains of pain on the probability of analgesic response. Univariable logistic regression models were created to determine odds ratios and 95% confidence intervals for prespecified variables of interest that could impact analgesic response. Multivariable logistic regression models were created to address confounding and to examine variables affecting probability of analgesic response and their adjusted odds ratios. All analyses were conducted using SAS Version 9.3 (SAS Institute, Cary, NC).

### Sample size calculation

We wanted to assess and compare characteristics of responders and nonresponders for the intervention of interest. This was an exploratory study, so we collected data on all patients who received ultrasound-guided local anaesthetic and steroid injections around II, IH, and GF nerves between January 1, 2009, and July 31, 2013 at our two pain centers. Data were included from records of patients who had neuropathic pain in the abdominal wall or groin following surgery or in association with inflammation of abdominal or pelvic viscera and where data on variables of interest were available for analysis.

## Results

**Figure 1.**
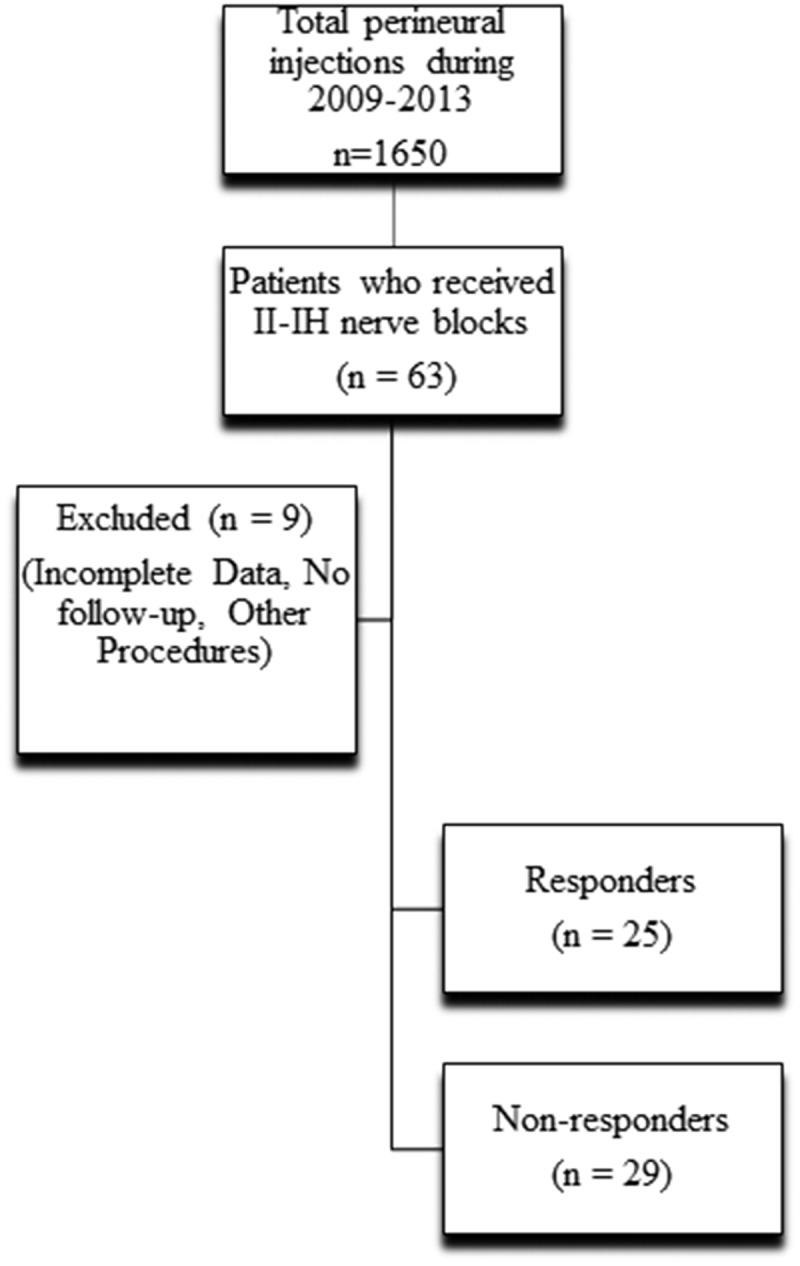
Flowchart of study selection inclusion and exclusion criteria and outcomes.

Sixty-three patients who received perineural injections around II and IH nerves at our clinics were identified but nine patients were excluded because of missing data points, lack of follow-up, and other procedures being performed in the interval between procedure and postprocedure follow-up at 6 weeks. Data from 54 patients (28 males and 26 females) were included for analysis in this study (Figure 1).

The mean intensity of pain was severe, with a median numerical rating score of 9 (interquartile range [IQR] = 8–10). All patients had described their pain using neuropathic descriptors (shooting, stabbing, hot-burning).^12^ Documented physical examination findings included evidence of allodynia, hyperalgesia, or hypoesthesia. Based on our criteria for analgesic response, 25 (46.3%) of these patients were identified as responders. Baseline characteristics of the cohort are presented in Tables 1 and 2. There were no significant differences between the two groups for age and gender distribution or site of pain.

**Table 1.** Patient demographics and morbidities associated with neuropathic pain. Values are median (IQR [range]), number (proportion), or mean (SD).

Characteristic	Responders (*n* = 25)	Nonresponders (*n* = 29)	*P* value
Age (years), mean (SD)	44.96 (14.60)	47.10 (14.62)	0.593
Sex (female/male) (%)	9 (36%)/16 (64%)	17/12 (59%/41%)	0.095
Pre–nerve block pain NRS score, median (IQR)	9.00 (8.00–10.00)	9.00 (8.00–10.00)	0.540
Pain laterality (unilateral/bilateral)	22 (88%)/3 (12%)	26 (90%)/3 (10%)	0.847
Pain site			
Lower abdominal wall, groin, medial thigh	18 (72%)	21 (73%)	0.248
Lower abdominal wall, groin, medial thigh, and genital area	7 (28%)	8 (27%)	
Other pain syndromes			
None	15 (60%)	20 (69%)	0.5134
Generalized pain	1 (4%)	3 (10%)	
Headache	2 (8%)	2 (7%)	
Lower limbs	7 (28%)	4 (14%)	
Surgery type			
Laparotomy, abdominal hysterectomy, or other pelvic surgery	10 (40%)	10 (34%)	0.301
Open inguinal or femoral hernia repair	6 (24%)	4 (14%)	
Other^a^	9 (36%)	15 (52%)	
Onset of severe pain in patients with CPSP			
Postoperative period: Immediate	8 (32%)	9 (31%)	0.1202
Postoperative: After 3 months but before 1 year	6 (24%)	2 (7%)	
Postoperative: After 1 year	2 (8%)	1 (3%)	
Duration of pain (months)	60.00 (36.00–96.00)	36.00 (24.00–72.00)	0.194
Duration of pain by categories			
More than 3 months but up to 1 year	1 (4%)	1 (3%)	0.139
More than 1 year but up to 5 years	8 (32%)	17 (59%)	0.049^b^
More than 5 years	16 (64%)	11 (38%)	0.054^c^
Depression (yes/no)	6 (24%)/19 (76%)	4 (14%)/25 (86%)	0.336
Anxiety (yes/no)	4 (16%)/21 (84%)	11 (38%)/18 (62%)	0.068
Diabetes mellitus (yes/no)	0 (0%)/25 (100%)	4 (14%)/25 (86%)	0.115
Abdominal or pelvic inflammatory conditions^d^ (yes/no)	4 (16%)/21 (84%)	7 (24%)/22 (76%)	0.1343

^a^Other: Laparoscopy surgery for removing adhesions.

^b^Comparing patients with history of 1–5 years of pain in both groups.

^c^Comparing patients with more than 5 years of pain in both groups.

^d^Inflammatory bowel disease (Crohn’s disease and ulcerative colitis)/endometriosis/interstitial cystitis.

IQR = interquartile range; NRS = Numerical Rating Scale; CPSP = chronic postsurgical pain.

**Table 2.** Univariable analysis to determine odds ratios and 95% confidence intervals for factors affecting analgesic success of perineural local anaesthetics and steroids (more than 30% reduction in pain 6 weeks after the procedures).

Variable	Odds ratio	95% Confidence interval	*P* value
Diabetes mellitus	<0.001	<0.001 to >0.999^a^	0.022
Male	2.518	0.837 to 7.576	0.095
Unilateral pain	0.846	0.155 to 4.623	0.847
Anxiety	0.312	0.084 to 1.151	0.068
Depression	1.974	0.487 to 7.994	0.336
Inflammatory disease	0.336	0.078 to 1.441	0.134

^a^Odds ratio could not be calculated because all patients who had diabetes mellitus also had no response to the intervention.

Ninety-six percent of patients (52 out of 54) had pain for more than one year and 50% of the patients reported pain for more than 5 years. A higher proportion of nonresponders than responders had pain for more than one year and up to 5 years (8/25 [32%] of responders and 17/29 [59%] of nonresponders; *P* = 0.049). However, there was no difference between proportions of responders and nonresponders who had pain for more than 5 years (16/25 [64%] of responders and 11/29 [38%] of nonresponders; *P* = 0.054; Table 1).

Thirty patients (64%) from the overall cohort had surgical procedures on the abdomen and pelvis prior to onset of persistent abdominal wall and groin pain (i.e., a diagnosis of CPSP) and the remaining 24 (36%) had pain in these locations secondary to abdominal or pelvic visceral inflammatory pathologies. Of the patients with CPSP, 32% of responders and 31% of nonresponders reported onset of severe pain in the immediate postoperative period, and the remainder reported an increase in intensity of pain after 3 months of surgery (Table 1). Operative procedures included laparotomy for bowel resection, abdominal hysterectomy, and open inguinal or femoral hernia repair. Only two responders and one nonresponder reported postoperative complications (local infection or hematoma). Twenty-four patients (36% of responders and 52% of nonresponders) had a diagnosis of one or more inflammatory etiologies (inflammatory bowel disease, endometriosis, or history of multiple operations) that suggested fibrosis or scarring as the possible etiology for nerve entrapment resulting in neuropathic pain.

Prevalence of conditions related to causation, progression, or worsening of neuropathic pain was extracted from the database and medical records. These conditions included diabetes mellitus, anxiety, and depression. Four out of 54 (7.5%) patients in the cohort had diabetes mellitus. There was a higher incidence of diabetes mellitus among nonresponders compared to responders (14% and 0%, respectively; *P* = 0.022). We screen for presence of anxiety and depression in our clinics by administering the Hospital Anxiety and Depression Scale (a score of 8 or higher on anxiety and depression components indicates the presence of these conditions; the maximum score is 21 for each of the two components).^27^ The prevalence of anxiety was 28% and the prevalence of depression was 19% in the overall cohort in this study. There was no significant difference between prevalence of anxiety and depression among responders and nonresponders (Tables 1 and 2).

We attempted to evaluate impact of three independent variables (gender, presence of anxiety, inflammatory bowel or gynecological pathologies) on the dichotomous outcome of presence or absence of analgesic response while controlling for confounding by performing multivariable logistic regression analysis. We used only three independent variables in our model to avoid overspecification and we chose these variables based on a combination of perceived clinical significance and results of our univariable analysis. The results of this analysis are presented in Table 3. We examined our model for collinearity among independent variables and we were able to rule it out on the basis of a low value for the variance inflation factors (between 1 and 1.1). None of the variables examined in our model had a significant impact on analgesic response. We ensured that our model fit the collected data by using the Hosmer-Lemeshow test for goodness of fit that was nonsignificant (*P* = 0.911), suggesting that the model fit was adequate, and by examining the *c*-statistic (= 0.73). This model was constructed in an attempt to provide exploratory insights and was not intended to be a robust explanatory model.^28^

**Table 3.** Multivariable regression analysis for factors associated with analgesic success of perineural local anaesthetics and steroids (more than 30% reduction in pain 6 weeks after the procedures).

Variable	Odds ratio	95% Confidence interval	*P* value
Female	0.25	0.05, 1.17	0.077
Anxiety	2.31	0.48, 11.17	0.299
Inflammatory disease	4.44	0.83, 23.78	0.081

Data regarding pharmacological therapies and perineural injections are reported in Table 4. Seventy-two percent of patients in both cohorts were taking one or more medications for treatment of neuropathic pain at the time of the perineural injection. Forty-eight percent of responders and 59% of nonresponders were on opioid medications and the median doses (in daily oral morphine equivalents in milligrams) were 16.5 mg (IQR = 0–90) and 30 mg (IQR = 18–180), respectively, with no significant difference between groups. In addition to receiving perineural injections of local anaesthetics and steroids around IH and II nerves, 19 patients in the study cohort (32% of responders and 36% of nonresponders) received perineural injection around the GF nerve because they had evidence of neuropathic pain in the sensory distribution of this nerve. No procedural-related complications or adverse effects during the follow-up period related to perineural injections of LA and steroids were reported.

**Table 4.** Baseline (pre–nerve block) analgesic medications and details of perineural injections. Values are number (proportion) or median (IQR [range]).

Characteristic	Responders (*n* = 25)	Nonresponders (*n* = 29)	*P* value
Ongoing neuropathic medications^a^ (yes/no)	18 (72%)	21 (72%)	0.9730
	7 (28%)	8 (28%)	
On opioids (yes/no)	12 (48%)	17 (59%)	0.4349
	13 (52%)	12 (41%)	
Daily opioid dose in oral morphine equivalents (mg), median (IQR)	16.5 (0.00–90.00)	30.00 (18.00–180.00)	0.3254
Type of nerve blocks			
II+IH	17 (68%)	18 (64%)	0.5803
II+IH+GF	8 (32%)	11 (36%)	

^a^Neuropathic medications include any combination of gabapentinoids, tricyclic antidepressants, and serotonin norepinephrine reuptake inhibitors.

IQR = interquartile range; II = ilioinguinal nerve; IH = iliohypogastric nerve; GF = genitofemoral nerve.

## Discussion

Chronic neuropathic pain in the abdominal wall and groin secondary to operative interventions or inflammatory conditions of the viscera is often of a high intensity and unresponsive to conventional recommended treatments. Our retrospective case series on this patient population revealed that ultrasound-guided perineural injections of LA and steroids around the II and IH nerves with or without injection around GF nerves provided analgesic benefit in almost every other patient (46.3% of subjects) with severe refractory neuropathic pain who received this intervention. The impact of duration of pain prior to the procedures on analgesic efficacy of this treatment was unclear. Univariable analysis revealed a decrease in efficacy of the procedures after pain had been present for one year as evidenced by a significantly higher number of nonresponders (compared to responders) in our study who had persistent pain for a year or longer but with no impact of duration on efficacy if pain had been present for less than one or more than 5 years. However, the effect of duration was not analyzed in multivariable analysis due to the smaller number of patients in the categories for this variable. This is the first reported case series of outcomes of ultrasound-guided perineural injections of LA and steroid around II, IH, and GF nerves in patients with chronic abdominal wall and groin neuropathic pain secondary to surgery or inflammatory visceral conditions.

Our study cohort included patients with CPSP and those with chronic abdomino-pelvic visceral inflammatory conditions but there was no difference between these two groups in incidence of analgesic success (52% and 48% of responders, respectively) with the study intervention. A recent scoping review on perineural steroids for the treatment of chronic post-herniorrhaphy groin pain^23^ identified four case series that reported analgesic efficacy of perineural steroids around II, IH, and GF nerves in patients with CPSP following inguinal herniorrhaphy.^18–21^ All of the studies reported an analgesic benefit with this intervention with a reduction in pain NRS scores by 55%–75%. However, there was heterogeneity in doses of steroids, number of injections (varying from one to seven), and interval between intervention and follow-up (one to 68 months). The analgesic benefit from these studies was higher than in our retrospective study. Possible reasons for this difference include higher doses of steroids, repeat procedures, and enrollment of patients with CPSP in these studies while excluding pain from inflamed viscera that can result in severe scarring/fibrosis around cutaneous nerves. All patients in our study cohort had no other injections until completion of the follow-up at 6 weeks after the study intervention.

In our study cohort, pain was diagnosed as neuropathic in its presentation by pain physicians at our clinic based on the descriptors used by the patients and the results of physical examination. Though it is ideal to use validated tools to screen for neuropathic pain,^29^ the diagnosis of neuropathic pain is essentially clinical.^30^ However, validated tools and investigations (e.g., Douluer Neuropathique 4, quantitative sensory testing), can help establish this diagnosis with greater accuracy while also helping to delineate the mechanism and potential treatment.^31^

The success rate for analgesia reported in our study (25 responders out of 63 subjects exposed to the treatment) is encouraging because pharmacological therapies recommended for neuropathic pain^13^ were ineffective for all of our patients who received perineural injections of LA and steroids. A high failure rate of pharmacological trials (number needed to reduce pain by 30%–50% varies from three to seven) has also been documented in other publications on this topic.^1–5^ The incidence of serious adverse effects with these medications (e.g., cognitive impairment, weight gain, autonomic disturbance) is also high.^12^ Chemical, radiofrequency, or surgical neurectomy is an analgesic option, but long-term pain relief is not assured and morbidity associated with these procedures is also a concern.^9^ Ultrasound-guided delivery of perineural LA and steroids has a low cost and incidence of adverse effects, and it can relieve neuropathic pain through multiple mechanisms.^15,16^

Though anatomic landmarks have often been used in the past for guiding II and IH nerve injections,^17,18^ this technique is unreliable in ensuring delivery of injectate around injured nerves.^33^ Imaging modalities that have been used for guiding these injections include computed tomography guidance and ultrasound.^19,20^ Ultrasound has been shown to enhance accuracy of perineural injections, it can be done in the pain clinic under sterile conditions, and it does not expose patients and health care providers to radiation. The ability to visualize muscle layers, blood vessels (the deep circumflex iliac artery is in close proximity to the II and IH nerves), peritoneum, and bowel enhances accuracy (and potentially efficacy) while minimizing the risk of inadvertent injury to neural and nonneural structures.^34^

Approximately half of the patients (53.7%) in our study cohort did not attain analgesic benefit from perineural steroids around II and IH nerves. A possible reason for failure to relieve pain in these patients is chronicity of pain because duration of pain is often inversely related with probability of analgesic success with perineural steroids, often due to the presence of dense fibrosis around the II and IH nerves in the fascial plane between the internal oblique and transrversus abdominis muscles or development of central sensitization.^34^ We chose to categorize duration of pain in three epochs (from 3 months up to one year, from one year up to 5 years, and more than 5 years) because chronic pain is usually accepted as the presence of pain for more than 3 months, patients with severe neuropathic pain are often referred to pain physicians after one year of onset of pain,^35^ and 5 years appears to be the inflection point for a sharp drop-off in incidence of positive analgesic outcomes following interventional treatments for neuropathic pain.^36^ This rationale is partially supported by our results that show that duration of pain longer than one year reduced the probability of analgesic benefit. Only 32% (8/25) of respondents compared to 59% (17/29) of nonrespondents had duration of pain of over a year (*P* = 0.049). However, we are unable to propose that duration of pain and probability of analgesic success of perineural interventions are inversely correlated because there was no difference in analgesic response in our subcohort of patients who had pain for more than 5 years. Further, the significance of lower incidence of response in subjects who had pain for more than one but less than 5 years is unclear because even a small change in the counts of responders and nonresponders would affect the analysis. More research with larger data sets is required to arrive at reliable conclusions regarding the impact of duration of pain to this intervention.

Another possible reason for analgesic failure with perineural steroids for peripheral neuropathic pain is the presence of diabetes mellitus, because diabetes can be associated with active axonal degeneration, ischemic injury, and microvasculitis.^37^ However, we do not have data on either the incidence of peripheral neuropathy or the severity of glycemic impairment in the diabetic patients in our study. Further, only 4 out of 29 patients among the nonresponders and none of the responders had diabetes. It is difficult to draw meaningful conclusions from these small numbers of patients and we do not recommend that perineural LA and steroid therapies should be withheld from patients with neuropathic pain who have diabetes. Finally, factors such as high grades of anxiety, depression, and nociceptive component of mixed peripheral pain syndromes can also reduce analgesic benefits from treatments for neuropathic pain,^38^ but we did not find these associations in our study.

We divided the patients in our cohort into responders and nonresponders based on the analgesic outcomes at 6 weeks after the intervention. We assess our patients at this time point because the peak effect of long-acting steroids manifests around 4 to 6 weeks after administration.^39^ Though our clinical protocols mandate postintervention assessment, we were unable to extract data for nine patients who did not return for follow-up. There may be several reasons for these missing data points, but lack of efficacy of the intervention is a possibility. A conservative assessment of beneficial effects of perineural steroids would lower the analgesic success rate from 46% (25/54) to 40% (25/63). This is still a promising outcome for a cohort that had limited options for relieving severe refractory neuropathic pain. However, a prospective, randomized controlled trial on patients with chronic neuropathic abdominal wall and groin pain with blinding of participants and observers is required to estimate the analgesic potential of perineural steroids at multiple time points (1, 3, 6, and 12 months) after the interventions. Recommendations for sample size and methodology for trials have also been provided in recently published reviews by our group.^23,41^

Analgesic response, the main outcome of this study, was dichotomous because it was based on patients reporting 30% or more reduction in their pain or not at the follow-up 6 weeks after the intervention. Though this cutoff is based on recommendations for assessment of efficacy of therapies for pain,^26^ lack of information on absolute pain scores after the intervention and absence of longitudinal, periodic follow-ups are limitations of this study. Categorization of continuous outcomes is efficient for understanding the impact of variables on the outcome, but it also limits the usefulness of the available data. We did not report absolute pain scores in this study because the earlier versions of our database allowed us to record dichotomous outcomes for analgesic therapies while not listing the absolute pain scores. We also recognize the potential for a type 1 error (finding a significant difference when none exists) due to multiple comparisons conducted in our univariate analysis. However, we did not find any significant differences between responders and nonresponders except for the difference in proportion of subjects with duration of pain between one and 5 years. Unrecognized confounding, responder bias, and missing data are also potential limitations of this retrospective study. However, retrospective designs may be useful in analgesic research by taking advantage of samples of convenience for development of pilot data that provide the basis for conducting high-quality randomized and controlled prospective studies.^40^ We also acknowledge that comparison of our study cohort with a group of patients managed with conventional medical management would have allowed an informed assessment of benefits and harms of perineural interventions. However, the referral criteria for our two clinics and the long wait times mandate that only patients who have failed to respond to first-, second-, and third-line pharmacological treatments and physical therapies (desensitization) for neuropathic pain and who continue to have moderate or severe pain are accepted for a trial of interventional approaches. There is also a robust body of published literature regarding success and failure rates of conventional medical management in peripheral neuropathic pain.^12,16,17^

Our results suggest that administration of perineural steroids can ameliorate chronic postsurgical and visceral inflammation related peripheral, severe refractory neuropathic pain in the abdominal wall and groin 6 weeks after the interventions in a significant number of patients.
